# Quantitative assessment of brain atrophy in bvFTD: Implications for diagnostic conversion

**DOI:** 10.1002/dad2.70307

**Published:** 2026-04-20

**Authors:** Chloe Beydoun, Halle Quang, Sophie Matis, Jessica L. Hazelton, James Carrick, Giorgio G. Fumagalli, Olivier Piguet, Ramon Landin‐Romero

**Affiliations:** ^1^ Sydney Medical School, Faculty of Medicine and Health The University of Sydney Sydney New South Wales Australia; ^2^ Brain & Mind Centre The University of Sydney Sydney New South Wales Australia; ^3^ Sydney School of Health Sciences, Faculty of Medicine and Health The University of Sydney Sydney New South Wales Australia; ^4^ School of Psychology, Faculty of Science The University of Sydney Sydney New South Wales Australia; ^5^ Center for Mind/Brain Sciences‐CIMeC University of Trento Trento Italy

**Keywords:** anterior cingulate cortex, anterior temporal cortex, disease progression, frontoinsular, frontotemporal dementia, medial temporal lobe, orbitofrontal cortex, visual rating scales

## Abstract

**INTRODUCTION:**

Brain atrophy increases diagnostic confidence in behavioral variant frontotemporal dementia (bvFTD); however, standardized tools have not been systematically applied. This study evaluated visual rating scales (VRS) to quantify atrophy in a longitudinal bvFTD cohort with differing diagnostic certainties.

**METHODS:**

Ninety‐three probable and 15 possible bvFTD patients were recruited. Five validated VRS were applied to magnetic resonance imaging scans by blinded raters. Receiver operating characteristic curves and logistic regression examined baseline differentiation and predictors of probable bvFTD. Longitudinal analyses and qualitative case review assessed VRS as predictors of diagnostic conversion.

**RESULTS:**

Bilateral orbitofrontal and frontoinsular atrophy differentiated probable from possible bvFTD (areas under the curve > 0.7). Baseline left orbitofrontal VRS strongly predicted baseline probable bvFTD (odds ratio = 3.77, *p *= 0.002). Converters to probable bvFTD (5/10 cases) had higher baseline left orbitofrontal VRS than non‐converters (Mann–Whitney *U* = 5, *p *= 0.053). Case reviews supported the quantitative findings.

**DISCUSSION:**

VRS enhance diagnostic certainty and may assist in monitoring bvFTD disease progression across clinical settings.

## Highlights

- Orbitofrontal and frontoinsular visual rating scales (VRS) differentiated probable from possible behavioral variant frontotemporal dementia (bvFTD).
- Baseline left orbitofrontal VRS strongly predicted likelihood of probable bvFTD.
- Converters from possible to probable bvFTD had higher left orbitofrontal VRS than non‐converters.
- VRS represent a systematic way to assess atrophy in bvFTD both within and across clinical settings.
- VRS used alongside routine clinical practice could improve diagnostic certainty and help with prognosis.

## 1 BACKGROUND

Behavioral variant frontotemporal dementia (bvFTD) is a younger‐onset dementia marked primarily by behavioral changes such as disinhibition, apathy, empathy loss, stereotypy, and hyperorality.^1^ The clinical, pathological, and genetic heterogeneity makes predicting bvFTD disease progression particularly challenging.^2^ BvFTD often mimics psychiatric disorders and other dementias,^3^, ^4^, ^5^ leading to initial misdiagnosis in 50% to 71% of cases.^6^, ^7^ Additionally, ≈ 50% of people may receive a revised diagnosis,^8^, ^9^ with some people labelled as non‐progressive “phenocopies.”^10^ Current consensus criteria define bvFTD along a continuum from “possible” to “probable” based on brain changes observed in frontal and/or anterior temporal regions using imaging (i.e., magnetic resonance imaging [MRI,] single‐photon emission computed tomography [SPECT], positron emission tomography [PET]).^1^ The lack of quantitative guidelines for assessing atrophy in bvFTD, however, limits diagnostic consistency across clinical settings.^11^ Here, we evaluated the utility of visual rating scales (VRS) for systematically assessing atrophy and improving diagnostic stratification.

VRS help to quantify structural brain changes on MRI in a standardized and accessible manner.^12^ These scales guide clinicians to specific MRI slices and provide ordinal scores for assessing regional atrophy.^13^ VRS can distinguish between healthy aging and neurodegeneration, as well as among neurodegenerative diseases.^12^ Five VRS targeting the orbitofrontal, anterior cingulate, anterior temporal, frontoinsular, and medial temporal regions^14^, ^15^ have previously demonstrated diagnostic utility in distinguishing FTD from psychiatric disorders,^16^ other dementias,^17^ and among genetic FTD subtypes.^18^ Higher VRS scores are related to greater frontotemporal lobar degeneration (FTLD) severity in bvFTD cases classified by underlying pathological certainty.^19^ Longitudinal VRS changes, however, have not been examined in a naturalistic, clinically observed cohort of sporadic bvFTD cases, nor within the framework of current consensus diagnostic criteria. Therefore, the clinical utility of VRS for staging disease progression and diagnostic stratification during life remains underexplored.

To address these gaps, we retrospectively reviewed a large cohort of bvFTD patients recruited through FRONTIER, a specialized younger‐onset dementia clinic at the University of Sydney. We aimed to assess whether region‐specific brain atrophy, measured using five established VRS (i.e., orbitofrontal, anterior cingulate, anterior temporal, frontoinsular, and medial temporal), could distinguish between possible and probable bvFTD at baseline. Next, we aimed to determine whether baseline atrophy in these regions could predict diagnostic conversion from possible to probable bvFTD over time. We hypothesized that atrophy would be more severe in probable than possible bvFTD, and that greater baseline atrophy in the five VRS regions in possible bvFTD cases would be associated with increased likelihood of progression.

## 2 METHODS

### 2.1 Participants

**FIGURE 1:**
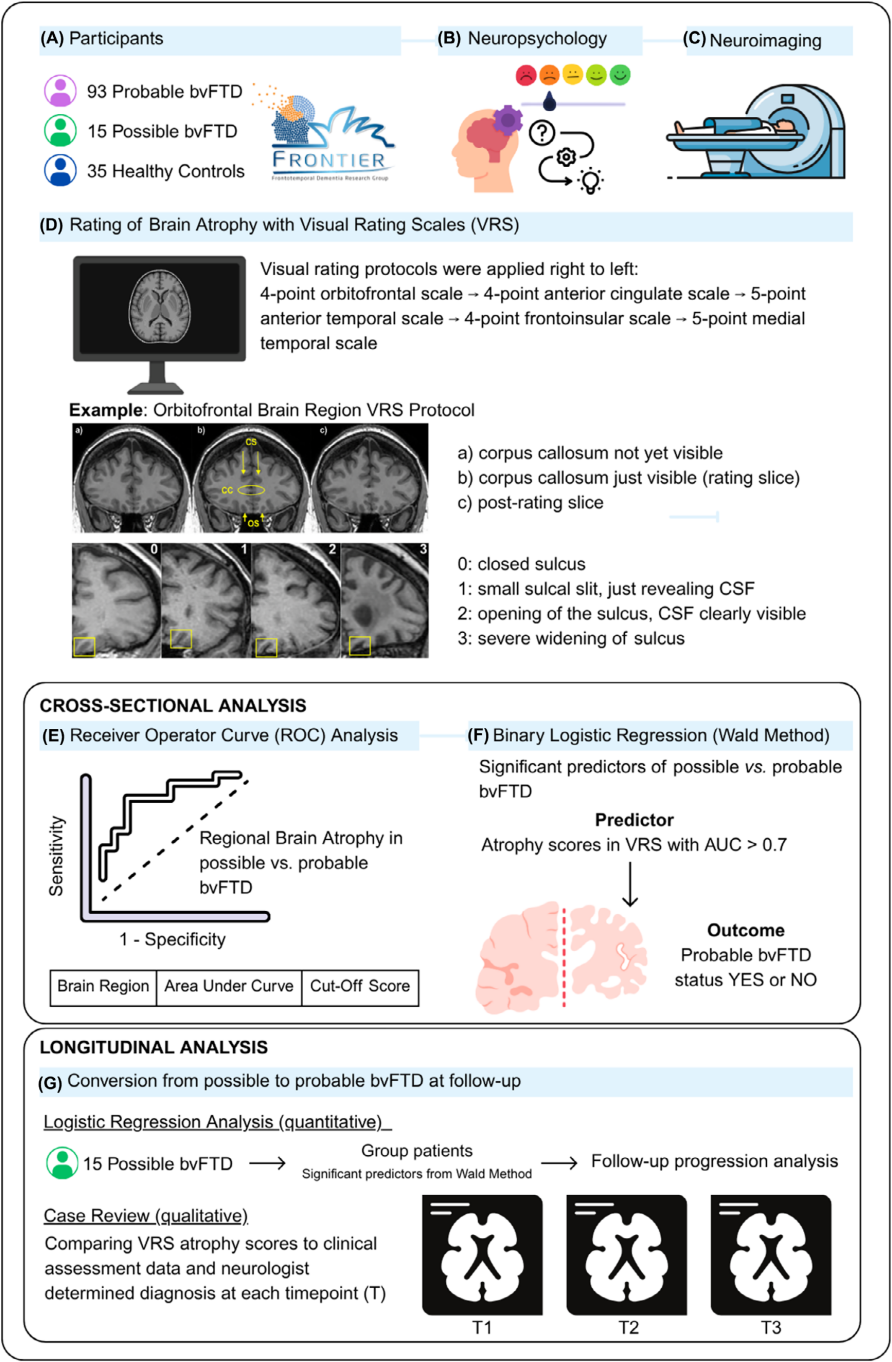
Experimental workflow diagram: (A) participants were recruited from FRONTIER database; (B) neuropsychological tests were conducted including cognition, executive functioning, and social cognition; (C) all participants had a 3D T1 MRI scan; (D) MRI scans were rated blind using VRS; (E) ROC analysis was performed for all atrophy scores for possible versus probable bvFTD; (F) binary logistic regression analysis (Wald method) was performed using ROC analysis atrophy score cut‐offs; (G) possible bvFTD participants were grouped according to significant predictors of atrophy from Wald method, and logistic regression analysis performed to determine conversion; the longitudinal analysis also included a qualitative case review of possible bvFTD participants with up to two annual follow‐up visits with MRI scans. bvFTD, behavioral variant frontotemporal dementia; CC, corpus callosum; CS, cingulate sulcus; MRI, magnetic resonance imaging; OS, olfactory sulcus; ROC, receiver operating characteristic; VRS, visual rating scales.

**FIGURE 2:**
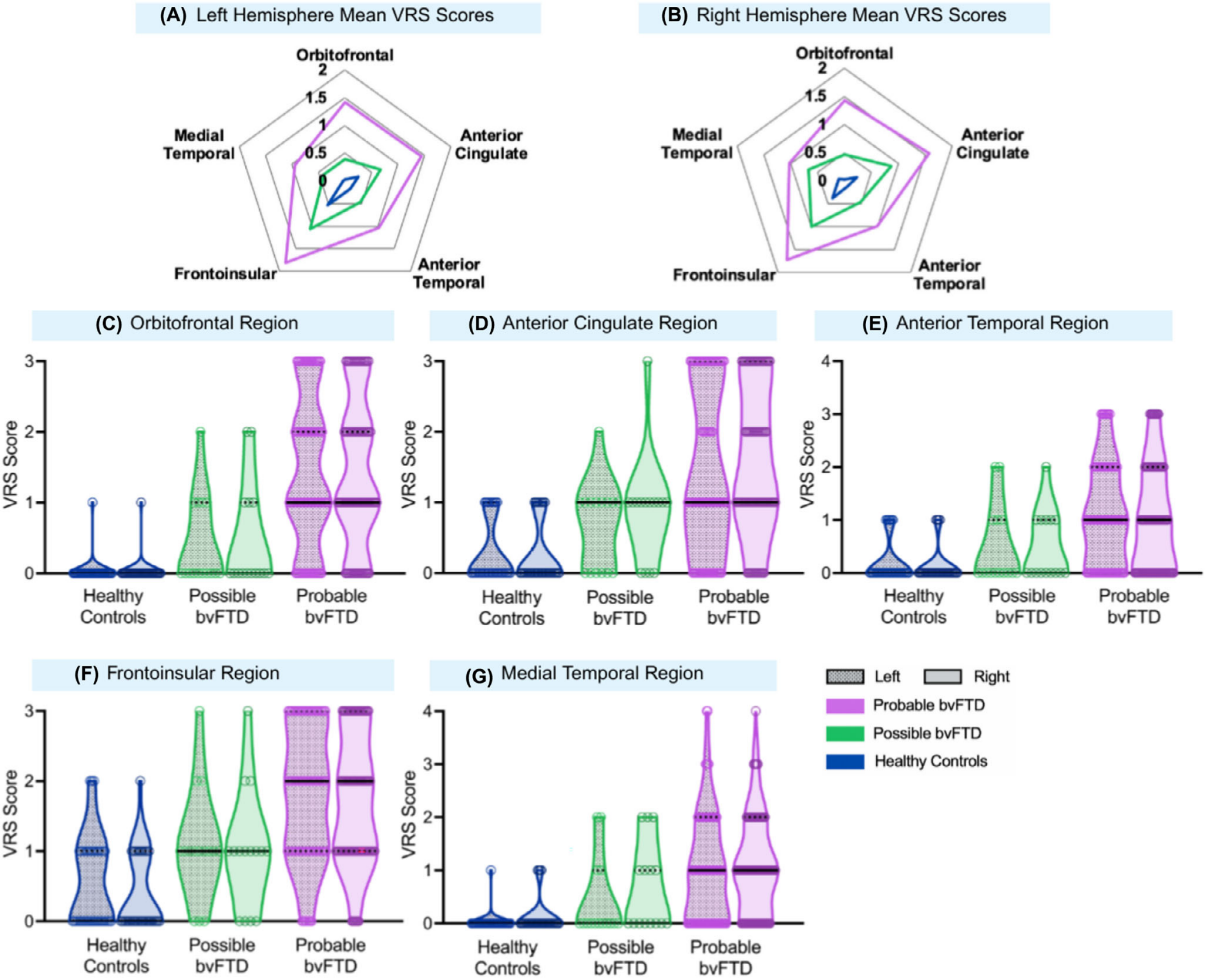
Visual rating scale (VRS) scores across patient groups. A, Radial plot of left hemisphere average VRS scores. B, Radial plot of right hemisphere average VRS scores. VRS scores in the bilateral (C) orbitofrontal region, (D) anterior cingulate region, (E) anterior temporal region, (F) frontoinsular region, (G) medial temporal region. bvFTD, behavioral variant frontotemporal dementia.

The current study included 108 individuals diagnosed with bvFTD through FRONTIER, the multidisciplinary frontotemporal dementia research clinic in Sydney, Australia. Diagnosis was established following current consensus criteria^1^ and was based on comprehensive clinical assessment with an experience behavioral neurologist, cognitive testing, informant reports, and structural brain MRI (see Figure 1 for the experimental workflow). This diagnostic process occurred prior to the VRS assessment used in the current study, which was performed independently by a rater blinded to diagnosis and supporting clinical information. No other imaging modalities (e.g., fluorodeoxyglucose PET), biomarkers, or pathological confirmation were available in the current cohort. Neuropsychological assessment included general cognitive examination using the Addenbrooke's Cognitive Examination‐III (ACE‐III)^20^ or Addenbrooke's Cognitive Examination‐Revised (ACE‐R),^21^ with statistical conversion to ACE‐III for comparison.^22^ For behavior and disease severity, measures included the Clinical Dementia Rating Scale for FTLD (CDR‐FTLD),^23^ Frontotemporal Dementia Rating Scale (FRS),^24^ Neuropsychiatric Inventory (NPI),^25^ and Cambridge Behavioural Inventory (CBI).^26^ Inclusion into the study required: (1) diagnosis of “possible” or “probable” bvFTD at initial presentation (Figure 1), (2) availability of T1‐weighted MRI suitable for VRS analysis, and (3) clinical assessment data within 3 months of MRI acquisition. Thirty‐five healthy control participants were selected from the FRONTIER volunteer database and local community clubs. Controls scored ≥ 88/100 on the Addenbrooke's Cognitive Examination (ACE‐R or ACE‐III),^27^ and 0 on the Sum of Boxes (SoB) score of the CDR‐FTLD.^23^ Exclusion criteria for all participants included the presence of concurrent primary psychiatric disturbance, other neurodegenerative conditions or neurological disorders, history of significant traumatic brain injury (with loss of consciousness > 5 minutes) and/or history of substance abuse.

The final dataset included 93 probable bvFTD patients, 15 possible bvFTD patients, and 35 healthy controls. Of the 15 possible bvFTD cases, 10 had at least one annual clinical and MRI follow‐up available. These cases were included in statistical analyses examining the predictive value of baseline atrophy patterns for diagnostic conversion. Furthermore, among these 10 cases, 6 had between two and three follow‐up visits. These were selected for a systematic qualitative case review conducted by a neurologist (C.B.), aimed at evaluating the predictive utility of VRS based on our baseline analysis within a real‐world clinical context.

All participants or their caregivers provided informed written consent for their clinical data to be used for research purposes in line with the Declaration of Helsinki. The study was approved by the ethics committee of the University of Sydney (2020/HE000408).

### 2.2 Structural MRI acquisition

A total of 152 T1‐weighted MRI scans were acquired using two equivalent 3T scanners, 143 baseline scans across all diagnostic groups, and 9 follow‐up scans from possible bvFTD patients (see Supplementary Materials in supporting information for scanner information).

### 2.3 Atrophy ratings

The lead author (C.B.) rated all participants’ MRI scans using five previously validated VRS, blinded to clinical information. These included a 5‐point anterior temporal scale,^28^, ^29^ a 5‐point medial temporal lobe scale,^30^ and 4‐point scales for the orbitofrontal, anterior cingulate, and frontoinsular regions^17^ (see Figure S1 in supporting information). Prior to rating, C.B. completed intra‐ and inter‐rater reliability training on an independent set of 30 MRI scans, using expert reference scores provided by a behavioral neurologist (G.F.) experienced in visual atrophy assessments (see Table S1 in supporting information). Internal consistency was excellent across all three rating trials, with Cronbach alpha coefficients of 0.928, 0.924, and 0.912 for trials 1, 2, and 3, respectively. Test–retest reliability demonstrated strong stability over time, with Pearson correlation coefficients > 0.7 (*p *< 0.001) for all comparisons. MRI scans were accessed via the XNAT open‐source imaging platform and rated across 1 mm slices in an anterior‐to‐posterior and right‐to‐left gradient, following standardized VRS protocols.

**RESEARCH IN CONTEXT**

1. **Systematic review**: The authors reviewed existing studies to evaluate the diagnostic value of visual rating scales (VRS) in behavioral variant frontotemporal dementia (bvFTD). Although neuroimaging evidence is a supporting feature of bvFTD diagnosis, standardized atrophy rating tools such as VRS have not been systematically applied across bvFTD cohorts with differing diagnostic certainty or followed longitudinally.
2. **Interpretation**: Findings demonstrate that VRS reliably distinguished probable from possible bvFTD based on frontotemporal atrophy. Baseline left orbitofrontal VRS predicted conversion from possible to probable bvFTD. Qualitative case reviews supported these results, and possible diagnostic pathways were identified. These results highlight the clinical relevance of VRS for enhancing diagnostic certainty and monitoring disease progression.
3. **Future directions**: Future research will require large longitudinal neurodegenerative disease cohorts, including presymptomatic cases, to investigate specificity and prognostic value of VRS. Feasibility of incorporating VRS into standardized clinical assessment protocols to improve diagnosis within and across clinical settings should be assessed.

### 2.4 Statistical analyses

Statistical analyses were conducted using SPSS (v29.0; SPSS Inc.). Group comparisons for continuous variables (e.g., age, education, disease duration, ACE‐III scores) were performed using one‐way analysis of variance (ANOVA) with Sidak post hoc tests to adjust for multiple comparisons. Categorical variables (e.g., sex distribution) were analyzed using chi‐squared tests. Independent samples *t* tests were used to compare clinical measures (e.g., disease duration, FRS Rasch scores, CBI, and NPI total scores) between possible and probable bvFTD groups. The statistical significance threshold was set at *p* < 0.05 for all analyses.

For cross‐sectional analysis, separate one‐way ANOVAs with Sidak post hoc testing were used to compare VRS atrophy scores across diagnostic groups (healthy controls, possible bvFTD, probable bvFTD) for each VRS. Receiver operating characteristic (ROC) curve analyses assessed the discriminative performance of each region in distinguishing possible from probable bvFTD. Regions with good discriminative ability (area under the curve [AUC] > 0.7) were entered into logistic regression models (Wald method) to identify the strongest predictors of probable bvFTD.

To evaluate prognostic utility, both quantitative and qualitative longitudinal approaches were applied. Binary logistic regression assessed diagnostic progression in possible bvFTD patients with follow‐up data, with the outcome defined as conversion to probable bvFTD. Predictor variables included presence (VRS ≥ 1) or absence (VRS = 0) of atrophy in regions identified as significant in cross‐sectional analyses. Additionally, a structured qualitative case review was conducted to examine correspondence between changes in VRS scores and neurologist‐determined diagnostic status (see Figure 1).

## 3 RESULTS

### 3.1 Participants

Demographic characteristics (age, sex, education) did not differ between groups at baseline (Table 1). Disease duration and CDR‐FTLD SoB scores were comparable between possible and probable bvFTD. The probable bvFTD group showed worse cognitive performance and greater functional impairment compared to the possible bvFTD group. Behavioral profiles between possible and probable bvFTD were broadly similar, though neuropsychiatric (NPI) and behavioral (CBI) scores showed substantial variability in both groups.

**TABLE 1:** Demographic, cognitive, and functional characteristics in possible bvFTD, probable bvFTD, and healthy controls.

	Possible bvFTD (*n* = 15)	Probable bvFTD (*n* = 93)	Healthy controls (*n* = 35)	*Statistic*	Post hoc
Age (years)	64.1 (8.3)	62.4 (8.0)	64.4 (3.8)	1.228^a^	–
Education (years)	12.6 (3.5)	12.5 (3.2)	13.3 (2.0)	0.870^a^	–
Sex (M:F)	12:3	58:35	18:17	3.698^c^	–
Disease duration (years)	4.2 (3.1)	4.5 (3.4)	—	0.192^b^	–
ACE‐III Total (/100)	86.6 (14.5)	72.9 (16.5)	95.7 (3.2)	34.205^***^	probable < possible < healthy controls
ACE‐III Attention (/18)	15.8 (3.3)	14.2 (3.4)	‐	0.886	‐
ACE‐III Memory (/26)	21.6 (5.9)	16.7 (6.1)	‐	0.631^**^	probable < possible
ACE‐III Fluency (/14)	9.4 (3.8)	5.5 (4.0)	‐	0.475^***^	probable < possible
ACE‐III Language (/26)	24.2 (2.0)	21.4 (4.9)	‐	9.621^*^	probable < possible
ACE‐III Visuospatial (/16)	14.8 (1.5)	13.4 (2.8)	—	2.997	‐
CDR‐FTLD SoB (/24)	4.46 (3.1)	5.99 (2.9)	—	0.351	–
FRS Rasch score	0.64 (1.8)	−0.77 (1.2)	—	3.082^***^	probable < possible
NPI Frequency	13.5 (7.3)	15.3 (6.7)	—	0.044	‐
NPI Severity	9.6 (6.1)	9.6 (4.2)	—	1.434	‐
NPI Total Score	25.8 (23.2)	26.3 (13.6)	—	3.223	‐
CBI Score	35.1 (20.4)	38.8 (15.6)	—	1.986	–

*Note*. Values are mean (standard deviation). Statistics represent.

Abbreviations: ACE‐III, Addenbrooke's Cognitive Examination‐III; bvFTD, behavioral variant frontotemporal dementia; CBI, Cambridge Behavioural Inventory; CRD‐FTLD SoB, Clinical Dementia Rating Scale for Frontotemporal Lobar Degeneration Sum of Boxes; FRS, Frontotemporal Dementia Rating Scale; NPI, Neuropsychiatric Inventory; ns, not significant.

^a^ one‐way analysis of variance,

^b^ independent sample *t* tests,

^c^ chi‐squared statistics,

* *p* < 0.05,

** *p* < 0.01,

*** *p* < 0.001.

Missing scores: probable bvFTD (2 education, 3 disease duration, 4 ACE‐III, 15 CDR‐FTLD SoB, 7 FRS, 10 NPI, 4 CBI), possible bvFTD (2 disease duration, 1 ACE‐III, 4 CDR‐FTLD SoB, 3 FRS, 5 NPI, 2 CBI).

### 3.2 Cross‐sectional analyses

#### 3.2.1 Group differences in atrophy scores

Group differences in VRS scores were observed across all brain regions of interest (Figure 2). The strongest effect sizes were observed in the bilateral orbitofrontal (OF) regions, with right OF (*F* [2, 140] = 34.822, *p *< 0.001, *η* ^2^ = 0.332, 95% confidence interval [CI; 0.205, 0.434]) and left OF (*F* [2, 140] = 35.041, *p* < 0.001, *η* ^2^ = 0.334, 95% CI [0.206, 0.435]). The right frontoinsular (FI) region also showed substantial differences (*F* [2, 140] = 33.973, *p* < 0.001, *η* ^2^ = 0.327, 95% CI [0.200, 0.429]), followed by right anterior cingulate (AC; *F* [2, 140] = 28.877, *p* < 0.001, *η* ^2^ = 0.292, 95% CI [0.167, 0.396]) and left FI (*F* [2, 140] = 27.785, *p* < 0.001, *η* ^2^ = 0.284, 95% CI [0.160, 0.388]). Smaller but significant differences were observed in left AC (*F* [2, 140] = 19.324, *p *< .001, *η* ^2^ = 0.216, 95% CI [0.101, 0.321]), bilateral anterior temporal (AT; left: *F* [2, 140] = 15.121, *p *< .001, *η* ^2^ = 0.178, 95% CI [0.071, 0.281], right: *F* [2, 140] = 14.063, *p* < .001, *η* ^2^ = 0.167, 95% CI [0.063, 0.270]), and bilateral medial temporal (MT; left: *F* [2, 140] = 15,921, *η* ^2^ = 0.185, 95% CI [0.077, 0.289], right: *F* [2, 140] = 16.845, *p* < .001, *η* ^2^ = 0.194, 95% CI [0.083, 0.298]) regions.

Post hoc Sidak testing revealed that the probable bvFTD group showed significantly higher atrophy scores compared to healthy controls across all regions bilaterally (all *p* < 0.001). The probable bvFTD group also showed significantly higher scores compared to the possible bvFTD group in OF (right: *p* < 0.001; left: *p* < 0.001), AC (right: *p* = 0.017; left: *p* = 0.018), FI (right: *p* = 0.007; left: *p* = 0.008), and left AT (*p* = 0.041) regions. In all other brain regions, differences in mean scores between possible and probable bvFTD were not significant, with a trend toward significance in the left MT (*p* > 0.06). The possible bvFTD group showed significantly greater atrophy in the right FI compared to controls (*p* = 0.05); no other regions differed significantly (all *p* values > 0.07).

#### 3.2.2 Diagnostic accuracy of atrophy scales at baseline

**FIGURE 3:**
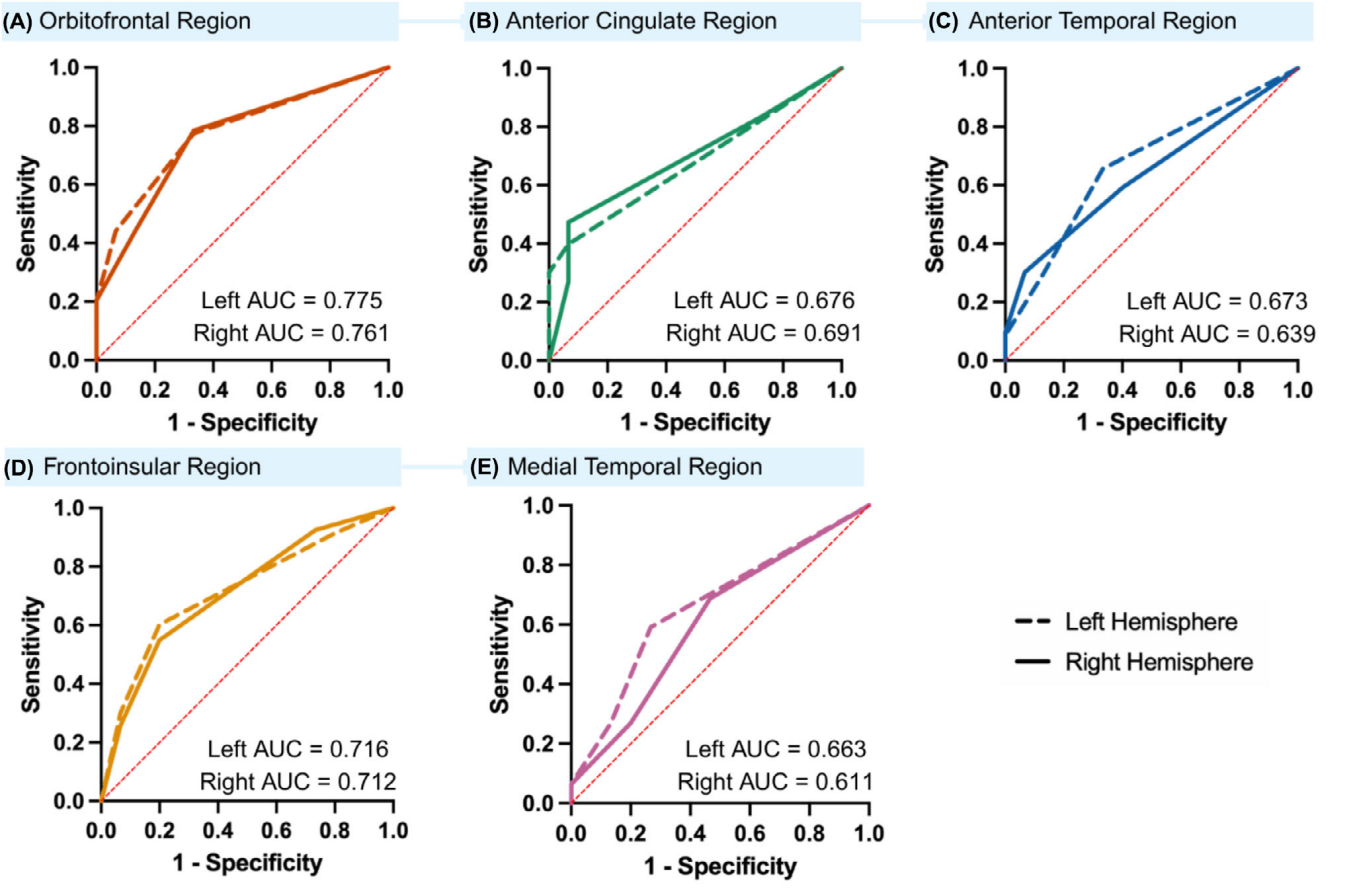
Receiver operator characteristic (ROC) analysis of VRS scores in distinguishing possible from probable bvFTD at baseline. ROC analyses in (A) orbitofrontal region, (B) anterior cingulate region, (C) anterior temporal region, (D) frontoinsular region, (E) medial temporal region. AUC, area under the curve; bvFTD, behavioral variant frontotemporal dementia; VRS, visual rating scales.

Figure 3 illustrates the diagnostic accuracy of regional atrophy measures in distinguishing possible from probable bvFTD at baseline. Sensitivity and specificity thresholds for probable bvFTD are detailed in Table S2 in supporting information. The OF and FI atrophy scores showed the strongest performance (left OF: AUC = 0.775, right OF: AUC = 0.761; left FI: AUC = 0.716, right FI: AUC = 0.712), with comparable sensitivity–specificity profiles.

Multiple logistic regression using Wald forward selection, restricted to regions with AUC > 0.7, identified left OF VRS as the strongest predictor of probable bvFTD (*B* = 1.825, standard error = 0.278, *p* < .001). Patients with left OF atrophy were 3.77 times more likely to be classified as probable bvFTD than possible bvFTD (95% CI: 1.61–8.84; *p* = 0.002). No other regions were included in the model based on the Wald forward selection method (right OF *p *= 0.455, right OF *p* = 0.411, left FI, *p* = 0.285).

### 3.3 Longitudinal analyses

#### 3.3.1 Quantitative atrophy‐based prediction of disease progression in possible bvFTD

We next examined whether orbitofrontal VRS scores at baseline related to diagnostic conversion at follow‐up. To do this analysis, 10/15 possible bvFTD patients that had at least one annual follow‐up appointment with a neurologist reported in the FRONTIER database were included. Among the 10 patients, 5 progressed to probable bvFTD, while 5 remained stable (4 retained the possible bvFTD diagnosis; 1 was reclassified as bvFTD phenocopy). Baseline left OF atrophy scores tended to be higher in those who progressed at follow‐up (mean rank = 7.00) compared to those who did not (mean rank = 4.00; Mann–Whitney *U* = 5.000, *p* = 0.053). To assess whether baseline left orbitofrontal atrophy predicted progression to probable bvFTD, binary logistic regression was attempted. The model did not converge, however, likely due to the small sample size. The near‐significant trend observed, however, in the Mann–Whitney *U* test (*p* = 0.053) suggests a preliminary signal, but larger samples are needed to confirm predictive value with adequate statistical power in quantitative analyses.

#### 3.3.2 Qualitative case review of longitudinal atrophy scores in possible bvFTD

A systematic case review was undertaken to assess the utility of atrophy scores in monitoring individual bvFTD trajectories within real‐world clinical settings. The sample comprised six possible bvFTD cases, each with more than one annual clinical and MRI follow‐up available. Demographic and clinical details are presented in Table 2, with individual VRS scores listed in Table S3 in supporting information. Diagnoses at each time point were compared against corresponding VRS scores and clinical assessments.

**TABLE 2:** Demographic and clinical characteristics of possible bvFTD patients with follow‐up.

	P01	P02	P03	P04	P05	P06
Age range (years)	74–76	58–60	46–48	73–75	52–54	71–73
Baseline disease duration (years)	2.12	4.15	4.17	6.87	3.46	6.01
Education (years)	17	11.5	11.5	9	12	8.5
Time (years) from baseline to:						
Follow‐up visit 1	2.51	1.16	1.01	5.31	1.05	1.08
Follow‐up visit 2	5.55	2.31	N/A	N/A	2.05	N/A
ACE‐III (/100)						
Baseline	89	90	98	95	90	94
Follow‐up visit 1	86	91	99	98	93	98
Follow‐up visit 2	82	91	N/A	N/A	93	N/A
Clinical diagnosis						
Baseline	Possible	Possible	Possible	Possible	Possible	Possible
Follow‐up visit 1	Probable	Probable	Possible	Possible	Probable	Possible
Follow‐up visit 2	Probable	Probable	N/A	N/A	Possible	N/A

Abbreviations: ACE‐III, Addenbrooke's Cognitive Examination‐III; bvFTD, behavioral variant frontotemporal dementia; N/A, not applicable; p, patient.

**FIGURE 4:**
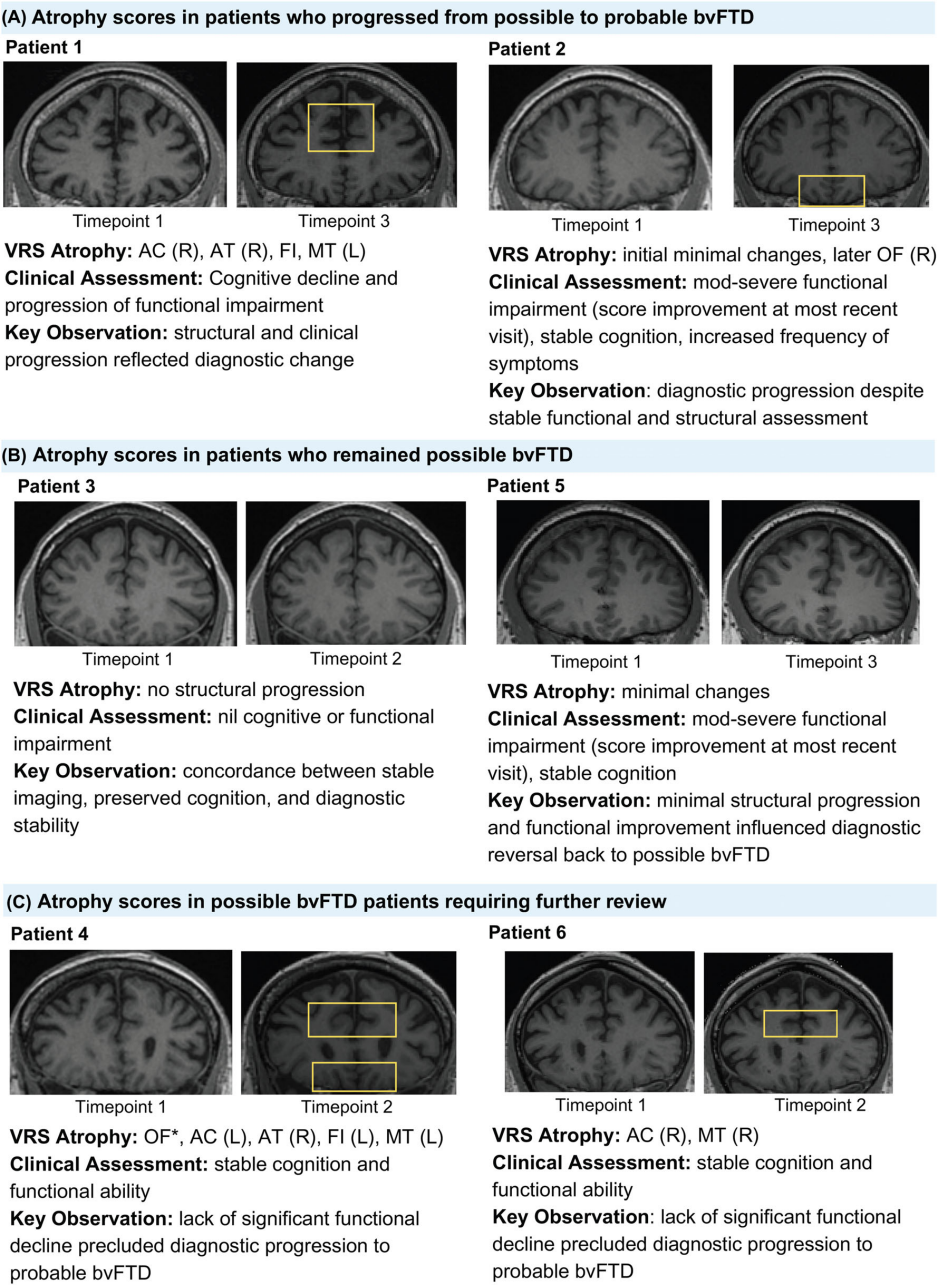
Case review of six possible bvFTD participants with longitudinal MRI scans and clinical assessment data. Imaging slices were selected from the time point when the most representative change occurred. A, Possible bvFTD patients who progressed to probable bvFTD at follow‐up. B, Possible bvFTD patients who remained possible bvFTD at follow‐up. C, Possible bvFTD patients with atrophy and clinical assessment warranting further review. * indicates VRS score increase by > 1 point. Yellow rectangles highlight regions were FRS changed per time point. AC, anterior cingulate; AT, anterior temporal; bvFTD, behavioral variant frontotemporal dementia; FI, frontoinsular; FRS, Frontotemporal Dementia Rating Scale; L, left; MRI, magnetic resonance imaging; MT, medial temporal; OF, orbitofrontal; R, right.

Our qualitative case review identified three distinct subgroups among possible bvFTD patients with follow‐up data (Figure 4). In group 1, which was diagnostic progression to probable bvFTD: P01 showed a prototypical trajectory, with concordant increases in atrophy scores, clinical severity, and diagnostic certainty. P02 progressed to probable bvFTD despite minimal structural change, in the context of moderate functional decline. Both cases had baseline left orbitofrontal atrophy, consistent with quantitative findings. In group 2, which consisted of those whose diagnosis remained possible bvFTD: P03 remained clinically and structurally stable. P05 was initially classified as probable bvFTD but later reclassified as possible bvFTD due to minimal atrophy and stable function. Group 3 patients consisted of those with indication for further review: P04 and P06 showed progressive atrophy on VRS but maintained cognitive and functional stability, likely precluding diagnostic conversion under current criteria. Notably, patients who did not progress clinically lacked baseline orbitofrontal atrophy, supporting its potential role as a biomarker of disease trajectory.

## 4 DISCUSSION

In the current study, we found that VRS‐derived atrophy scores demonstrated acceptable diagnostic accuracy in differentiating possible from probable bvFTD. Notably, orbitofrontal and frontoinsular atrophy were more prominent in probable bvFTD at baseline, while temporal lobe involvement was comparable across diagnostic groups. Baseline left orbitofrontal atrophy also showed potential as a predictor of diagnostic conversion from possible to probable bvFTD. Our qualitative case review further illustrated how VRS may support clinical decision making by identifying three distinct progression patterns, with practical implications for monitoring and diagnosis. These findings support the clinical utility of VRS as a simple, scalable tool for assessing disease severity and progression in real‐world clinical settings.

Current consensus criteria for probable bvFTD require neuroimaging evidence of frontotemporal atrophy,^1^ yet specific cut‐offs and typical atrophy patterns remain undefined. Our findings demonstrate that VRS are reliable tools that enhance diagnostic certainty at initial clinical presentation, in line with recent evidence.^12^ Probable bvFTD patients showed greater atrophy across all regions compared to possible bvFTD, suggesting a more advanced disease.^31^ Presence of prominent frontal involvement, particularly in the frontoinsular and anterior cingulate regions, aligns with prior autopsy‐confirmed studies.^32^ Importantly, our work suggests that frontal lobe atrophy scores via VRS provide a neuroanatomical signature in vivo that improves diagnostic confidence in probable bvFTD. Further, VRS provide a translatable and clinically reliable tool to assess brain atrophy, which doesn't rely on highly specialized computational neuroimaging skills.^11^ While computational analyses to predict single‐subject disease trajectories based on neuroimaging are becoming increasingly possible,^33^ these automated analyses are not available in many clinical environments, particularly in rural, regional, or resource‐limited settings, or in many low‐ and middle‐income countries. Additionally, these tools are often computationally intensive and require specialized infrastructure, limiting their feasibility for assessing individual patients in current routine practice. Further, these methods are currently limited by small sample sizes, limited clinical information, and binary model classifications resulting in poor generalizability in real‐world settings.^33^ Therefore, the use of VRS in routine neurological assessment provides a practical and promising avenue for the standardization of atrophy assessment across diverse clinical settings and may be a useful neuroimaging biomarker for future clinical trials.

A key finding from the longitudinal component of our study was that the severity of orbitofrontal atrophy at initial presentation was predictive of progression from possible to probable bvFTD longitudinally. While identifying atrophy in possible bvFTD cases may appear to challenge current diagnostic criteria,^2^ prior studies using VRS have similarly detected subtle atrophy in patients with low diagnostic certainty.^17^ Rather than contradicting consensus guidelines, our findings suggest that cortical changes may be missed during routine, unstructured MRI reviews or may vary between clinicians. Orbitofrontal degeneration can be expected in early bvFTD, given its strong association with personality and social functioning.^34^, ^35^ Beyond VRS, orbitofrontal atrophy has also been linked to disease progression in neuroimaging studies,^36^, ^37^ correlating with increasing severity scores over time.^38^ Our data further support the specificity of orbitofrontal atrophy. While low‐grade atrophy in temporal regions is occasionally seen in healthy aging controls using VRS, only one control in our cohort showed minimal orbitofrontal involvement. Indeed, subtle frontotemporal brain changes can occur as part of healthy aging;^39^, ^40^ however, these changes are often not detectable on routine clinical review. This finding suggests that orbitofrontal atrophy may also help distinguish bvFTD pathology from normal aging and may be supported by VRS. Additionally, prior studies have shown that orbitofrontal involvement is useful in differentiating bvFTD from other FTD subtypes, such as semantic dementia.^41^ Together, these results support orbitofrontal VRS measures as a clinically relevant tool for early bvFTD diagnosis and patient stratification in behavioral neurology.

Evidence of functional decline alongside progressive structural brain atrophy forms the basis of current consensus criteria for bvFTD probable diagnosis.^2^ Integrating structural and clinical data is essential for diagnostic accuracy: over‐reliance on imaging findings may lead to false positives, while typical bvFTD behaviors can occur in the absence of neurodegeneration.^28^ In our qualitative review of individual bvFTD diagnostic trajectories, patients P04 and P06 highlight this clinical–radiological balance. Both patients showed progressive atrophy on MRI but retained a diagnosis of possible bvFTD due to stable functional profiles, suggesting potential protective factors delaying phenotypic expression and warranting close follow‐up. In contrast, patients P01 and P03 followed clinical trajectories consistent with consensus criteria; P01 progressed from possible to probable bvFTD, with brain changes consistent with typical atrophy progression into contralateral (left to right) and posterior regions.^37^ P03 showed no clinical or structural progression, consistent with a slow and stable disease course that supported maintaining a diagnosis of possible bvFTD. Patient P05 presented a complex course, initially progressing to probable bvFTD before being reclassified as possible bvFTD due to the lack of progression in atrophy patterns, despite persistent moderate to severe functional impairment. This diagnostic uncertainty was supported by our VRS findings, underscoring its value in longitudinal MRI comparisons. Patient P02 also progressed to probable bvFTD despite minimal structural and clinical change, with elevated caregiver burden possibly influencing diagnostic decisions. This discordance may reflect subcortical or cerebellar pathology, a profile not captured by our five‐region VRS.^42^, ^43^ Overall, our qualitative case review demonstrates the clinical utility of integrating VRS‐derived atrophy scores with clinical assessments, not as standalone diagnostic tools, but as part of a comprehensive evaluation strategy to improve early detection, clarify diagnostic uncertainty, and guide follow‐up in bvFTD.

Our findings contribute to the limited body of research investigating the utility of VRS in the clinical diagnosis of bvFTD. While previous work has shown that summation of VRS scores across regions can differentiate high‐ and low‐confidence bvFTD cases,^19^ the strength of our study lies in its alignment with current consensus diagnostic criteria, enhancing its applicability to behavioral neurology practice. Importantly, we assessed the diagnostic sensitivity of each VRS region individually, rather than assuming equal contribution across regions. This approach improves tracking of individual disease trajectories and highlights the clinical relevance of regional atrophy patterns. Our analysis relied on demographic, clinical, and imaging data typically available at the first clinical encounter, supporting the feasibility of VRS implementation in routine diagnostic workflows, including cases in which different specialists may be reviewing the same patient. Our findings offer a foundation for improving diagnostic consistency across culturally diverse clinical settings and research centers. By using a simple, structured VRS approach aligned with consensus criteria, clinicians can apply a standardized method for assessing bvFTD‐related atrophy, reducing variability in interpretation and enhancing diagnostic harmonization globally.^38^ Furthermore, orbitofrontal VRS scores may serve as potential enrichment markers or stratification tools in clinical trials, helping to identify patients at higher risk of progression and improving trial design for disease‐modifying therapies.

Several limitations warrant consideration. First, the specialized nature of the FRONTIER FTD clinic, combined with known diagnostic and referral delays due to clinical heterogeneity and uncertainty,^44^ resulted in a cohort skewed toward mid‐ and later‐stage presentations. To mitigate this consideration, we matched both bvFTD groups for symptom duration. This matching, however, may have contributed to the lack of observed differences in temporal lobe atrophy. Indeed, temporal lobe atrophy was similarly low across diagnostic groups, echoing previous reports of minimal temporal involvement across bvFTD severity stages.^45^, ^46^, ^47^ This pattern suggests our cohort may predominantly reflect a frontal subtype of bvFTD, in which temporal atrophy influences early symptoms but not necessarily diagnostic progression.^48^, ^49^ Second, despite including one of the largest bvFTD cohorts to date, the number of probable versus possible bvFTD cases was unequal. Nonetheless, moderate to large effect sizes suggest the findings remain clinically meaningful. Future studies will benefit from larger samples of possible bvFTD with additional follow‐up, and the inclusion of presymptomatic individuals to strengthen predictive analyses of conversion. Technical limitations include minor variability in MRI contrast and potential lateralization bias when scoring both hemispheres simultaneously using VRS. Future studies could address this by mirroring left hemispheres onto the right and scoring each hemisphere independently and blindly, enhancing reliability and reducing scoring bias. Finally, although raters were blinded to participants’ group membership while rating images, the three possible groups of participants (i.e., control, possible, or probable bvFTD) were known to the rater a priori and introduces potential circularity. Future works including a range of neurodegenerative conditions, other differential diagnoses (i.e., late‐onset primary psychiatric illness, phenocopy syndrome) combined with healthy aging in large longitudinal cohorts will be useful to determine the specificity and sensitivity of the results in bvFTD. This work will be particularly useful to address the potential subtle atrophy observed in healthy aging.

In summary, our findings highlight the importance of region‐specific VRS atrophy scores, particularly orbitofrontal changes, in predicting diagnostic progression from possible to probable bvFTD. The strong performance and practical applicability of VRS protocols underscore their potential to enhance diagnostic consistency in clinical settings. To further enhance early bvFTD diagnosis and improve prognostic accuracy, future research should focus on developing standardized rating protocols and consensus guidelines for assessing brain atrophy. These tools should be implemented alongside current diagnostic criteria to support more consistent and reliable clinical evaluations.

## CONFLICT OF INTEREST STATEMENT

The authors declare no conflicts of interest.

## CONSENT

All participants or their caregivers provided informed written consent for their clinical data to be used for research purposes in line with the Declaration of Helsinki. The study was approved by the ethics committee of the University of Sydney (2020/HE000408).

## Supporting information



Supporting Information

Supporting Information
