# Supplementary material for: Quantitative assessment of brain atrophy in bvFTD: Implications for diagnostic conversion
Source: Alzheimers Dement (Amst). 2026 Apr 20;18(2):e70307. doi: 10.1002/dad2.70307 (PMC13095860; doi:10.1002/dad2.70307)
Supplement: Supplementary file 2 — Supporting Information [file DAD2-18-e70307-s002.docx]

**Supplementary Materials**

**Supplementary Methods**

**Scanner information**

Scans were obtained on either a Philips 3T scanner (n = 92; 61%) or a GE Discovery MR750 scanner (n = 60; 39%) using equivalent acquisition protocols with the following parameters; standard eight-channel head coil and harmonised protocols: 256 × 256 matrix, 200 slices, 1 mm slice thickness, 1 × 1 mm in-plane resolution, echo time/repetition time = 2.6/5.8 ms, and flip angle α = 8 (28).


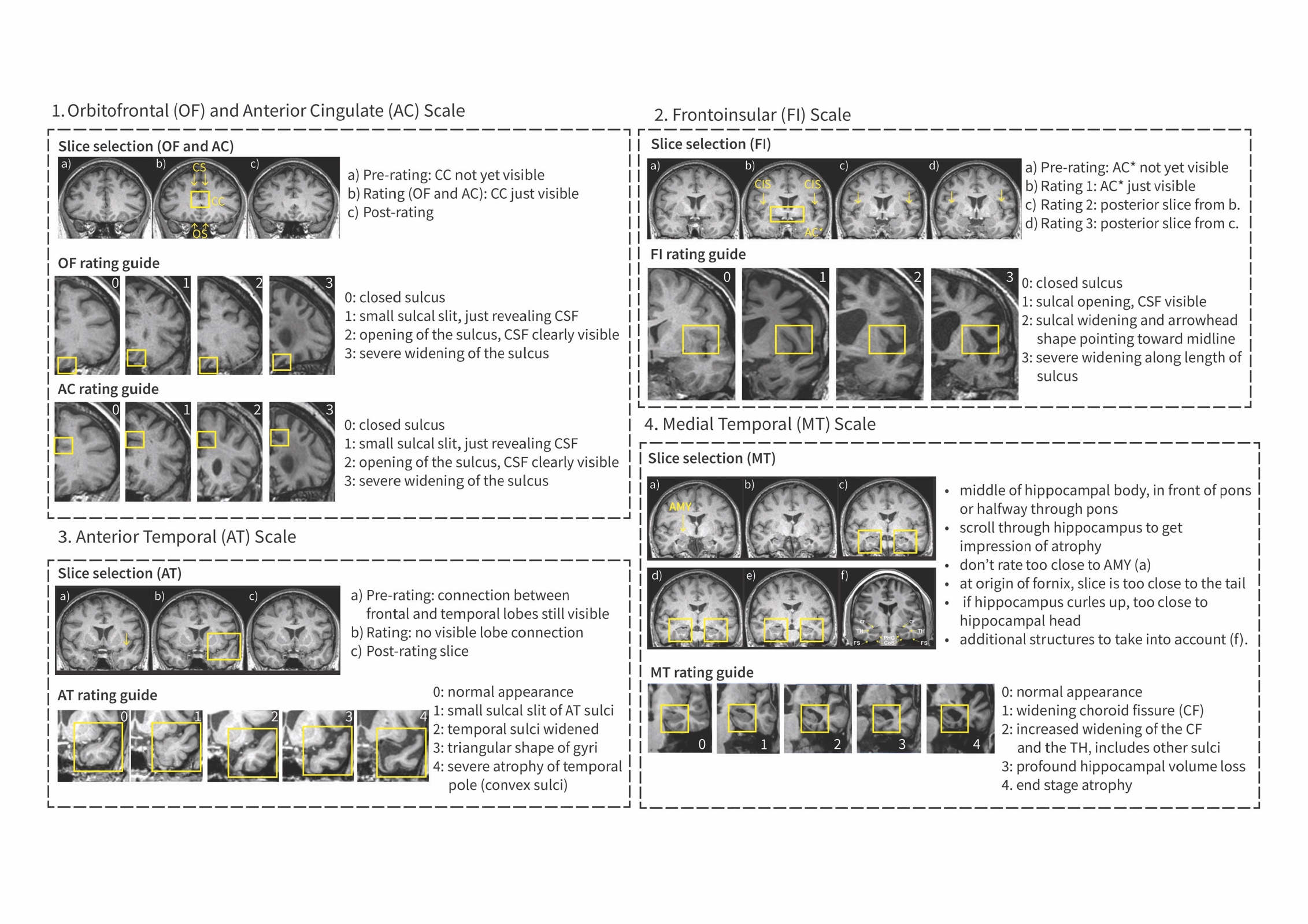


Supplementary Figure 1. Example of VRS protocol for behavioural variant frontotemporal dementia (bvFTD) adapted from (13) reproduced under the Creative Commons Attribution License (http://creativecommons.org/licenses/by/4.0/) (17). Each panel describes slice selection followed by rating guides for each VRS. Panel 1: Orbitofrontal (OF) and Anterior Cingulate (AC) scale. Panel 2: Frontoinsular scale. Panel 3: Anterior Temporal scale. Panel 4: Medial Temporal Scale. Abbreviations: AC: Anterior Cingulate; AC*: Anterior commissure; AT Anterior Temporal; AMY: Amygdala; CC: Corpus callosum; CIS: Circulus Insular sulcus; CS: Cingulate sulcus; CF: Choroid Fissure; CoS: Collateral sulcus; CSF: Cerebrospinal Fluid; FS: Fusiform sulcus; OS: Olfactory sulcus; PHG: Parahippocampal gyrus; TH: Temporal horn.

Supplementary Table 1. All correlations calculated using Spearman’s rank-order correlation coefficient (ρ) for ordinal data.

|  | Trial 1 | | Trial 2 | | Trial 3 | |
| --- | --- | --- | --- | --- | --- | --- |
| Brain Region | ρ | *p* | ρ | *p* | ρ | *p* |
| OF L | 0.907 | <0.001 | 0.940 | <0.001 | 0.873 | <0.001 |
| OF R | 0.776 | <0.001 | 0.867 | <0.001 | 0.746 | <0.001 |
| AC L | 0.515 | 0.003 | 0.506 | 0.003 | 0.584 | <0.001 |
| AC R | 0.610 | <0.001 | 0.671 | <0.001 | 0.590 | <0.001 |
| AT L | 0.473 | <0.001 | 0.548 | 0.002 | 0.474 | 0.008 |
| AT R | 0.541 | 0.002 | 0.483 | 0.007 | 0.499 | 0.005 |
| FI L | 0.607 | <0.001 | 0.629 | <0.011 | 0.547 | 0.002 |
| FI R | 0.547 | 0.002 | 0.545 | 0.002 | 0.677 | <0.001 |
| MT L | 0.508 | 0.004 | 0.459 | 0.011 | 0.541 | 0.002 |
| MT R | 0.633 | <0.001 | 0.658 | <0.001 | 0.397 | 0.03 |

Note. Inter-rater reliability results assessed between two independent raters (CB, GF) across three rating trials. *Abbreviations*: MT, medial temporal; AT, anterior temporal; OF, orbitofrontal; AC, anterior cingulate; FI, frontoinsular; L, left; R, right.

Supplementary Table 2. ROC Analysis Performance Metrics for Visual Rating Scales in possible vs probable behavioural variant frontotemporal dementia.

| Brain Region |  | AUC | Cut-Off Score | Sensitivity (%) | Specificity (%) |
| --- | --- | --- | --- | --- | --- |
| Orbitofrontal | L  R | 0.775  0.761 | 0.5  0.5 | 77  79 | 67  67 |
| Anterior Cingulate | L  R | 0.676  0.691 | 1.5  1.5 | 40  47 | 93  93 |
| Anterior Temporal | L  R | 0.673  0.639 | 0.5  0.5 | 66  59 | 67  60 |
| Frontoinsular | L  R | 0.716  0.712 | 1.5  1.5 | 60  55 | 80  80 |
| Medial Temporal | L  R | 0.663  0.611 | 0.5  0.5 | 59  69 | 73  53 |

*Note. Abbreviations*: AUC, area under the curve. L = Left; R = Right.

Supplementary Table 3. Longitudinal visual rating scale scores of six possible bvFTD patients with follow-up.

| PID | Visit | Clinical Diagnosis | OF R | OF L | AC R | AC L | AT R | AT L | FI R | FI L | MT R | MT L |
| --- | --- | --- | --- | --- | --- | --- | --- | --- | --- | --- | --- | --- |
| P01 | Baseline | Possible | 2 | 2 | 1 | 1 | 0 | 1 | 1 | 1 | 2 | 0 |
|  | Follow-up visit 1 | Probable | 2 | 2 | 1 | 1 | 0 | 0 | 1 | 2 | 1 | 0 |
|  | Follow-up visit 2 | Probable | 2 | 1 | 2 | 1 | 1 | 1 | 2 | 2 | 2 | 1 |
| P02 | Baseline | Possible | 0 | 1 | 0 | 0 | 0 | 0 | 0 | 0 | 1 | 0 |
|  | Follow-up visit 1 | Probable | 0 | 0 | 0 | 0 | 0 | 0 | 0 | 0 | 0 | 0 |
|  | Follow-up visit 2 | Probable | 1 | 1 | 0 | 0 | 0 | 0 | 0 | 0 | 1 | 0 |
| P03 | Baseline | Possible | 0 | 0 | 1 | 0 | 1 | 0 | 0 | 1 | 0 | 0 |
|  | Follow-up visit 1 | Possible | 0 | 0 | 0 | 0 | 0 | 0 | 0 | 1 | 0 | 0 |
| P04 | Baseline | Possible | 0 | 0 | 1 | 1 | 0 | 1 | 1 | 1 | 1 | 0 |
|  | Follow-up visit 1 | Possible | 2 | 1 | 1 | 2 | 1 | 1 | 1 | 2 | 1 | 1 |
| P05 | Baseline | Possible | 0 | 0 | 0 | 0 | 0 | 0 | 1 | 0 | 0 | 0 |
|  | Follow-up visit 1 | Probable | 0 | 0 | 0 | 0 | 0 | 0 | 1 | 0 | 0 | 0 |
|  | Follow-up visit 2 | Possible | 0 | 0 | 0 | 0 | 0 | 0 | 1 | 0 | 0 | 0 |
| P06 | Baseline | Possible | 0 | 0 | 0 | 1 | 0 | 0 | 1 | 2 | 1 | 1 |
|  | Follow-up visit 1 | Possible | 0 | 0 | 1 | 0 | 0 | 0 | 1 | 1 | 2 | 1 |

*Note. Abbreviations*: P, patient; R, right; L, left; OF, orbitofrontal; AC, anterior cingulate; AT, anterior temporal; FI, frontoinsular; MT, medial temporal
